# Climate and the biotic community structure plant resistance across biogeographic groups of yellow monkeyflower

**DOI:** 10.1002/ece3.9520

**Published:** 2022-11-22

**Authors:** Michael C. Rotter, Kyle Christie, Liza M. Holeski

**Affiliations:** ^1^ Department of Biological Sciences Northern Arizona University Flagstaff Arizona USA; ^2^ Department of Biology Utah Valley University Orem Utah USA; ^3^ Department of Plant Biology Michigan State University East Lansing Michigan USA

**Keywords:** climate, herbivore species richness, landscape, monkeyflower, phytochemical resistance, plant defense, plant species richness

## Abstract

Characterizing correlates of phytochemical resistance trait variation across a landscape can provide insight into the ecological factors that have shaped the evolution of resistance arsenals. Using field‐collected data and a greenhouse common garden experiment, we assessed the relative influences of abiotic and biotic drivers of genetic‐based defense trait variation across 41 yellow monkeyflower populations from western and eastern North America and the United Kingdom. Populations experience different climates, herbivore communities, and neighboring vegetative communities, and have distinct phytochemical resistance arsenals. Similarities in climate as well as herbivore and vegetative communities decline with increasing physical distance separating populations, and phytochemical resistance arsenal composition shows a similarly decreasing trend. Of the abiotic and biotic factors examined, temperature and the neighboring vegetation community had the strongest relative effects on resistance arsenal differentiation, whereas herbivore community composition and precipitation have relatively small effects. Rather than simply controlling for geographic proximity, we jointly assessed the relative strengths of both geographic and ecological variables on phytochemical arsenal compositional dissimilarity. Overall, our results illustrate how abiotic conditions and biotic interactions shape plant defense traits in natural populations.

## Graphical abstract

We find drastic differences in the relative strength of the effects that abiotic and biotic variables have on herbivory resistance arsenals. At least at the resolution of our available data, abiotic factors related to temperature and seasonality, and biotic factors associated with neighboring plant communities, play an outsized role in predicting resistance arsenal similarity compared with precipitation regimes or herbivore communities.

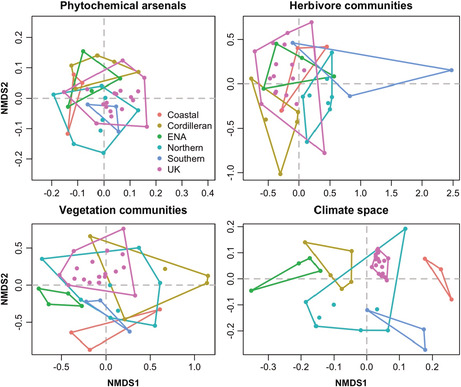

## 1 INTRODUCTION

Plants produce a diversity of phytochemical traits that act individually, additively, or synergistically in resistance of herbivory. Trait values in modern populations are shaped by past and current ecological interactions and evolutionary processes, including selection from multiple biotic and abiotic sources, and demographic processes such as genetic drift and gene flow (Linhart & Grant, 1996; López‐Goldar et al., 2020). While the study of within‐population defense traits and across‐population patterns of defense production both has a strong literature, connections between these two scales are logistically and statistically difficult and are relatively rare.

For a particular plant population, patterns of defense trait production may be influenced by resource availability and allocation strategy as well as selection from non‐resource‐related aspects of the abiotic and biotic environment. Within populations, optimal defense theory, the resource allocation hypothesis, and others, predict patterns of resource allocation to defense trait production (Coley et al., 1985; Hahn & Maron, 2016; Herms & Mattson, 1992; López‐Goldar et al., 2020). Trade‐offs between investment in defense and other aspects of life history strategy and/or the cost–benefit ratio of the defense investment underlie these hypotheses. Likewise, multiple hypotheses have been developed to explain the evolution and ecological consequences of within‐population phytochemical diversity such as the synergy hypothesis, the interaction diversity hypothesis, the moving target hypothesis, and the plant community variability hypothesis (Wetzel & Whitehead, 2019). Despite this well‐developed literature, studies of genetic‐based variation in defense and defense evolution within populations are often not well connected to broader patterns of evolution across a species range.

As with intra‐population studies, geographic patterns of inter‐population differences in plant defense have been investigated for decades. Despite the large number of publications describing defense production across elevational or latitudinal gradients, these studies are often at the inter‐species level (e.g., Coley & Aide, 1991; Levin, 1976; Moles et al., 2011; Rasmann & Agrawal, 2011; but see Anstett et al., 2015; Hahn et al., 2019). Thus, critical mechanistic links between the evolution of defense traits at the population level and defense trait similarity across landscapes remain unanswered. Climatic factors, resource availability, herbivore pressure, and the composition of herbivore communities and associated vegetative communities have all been shown to influence plant defense; these variables often co‐vary but to different extents and at different spatial scales (Hunter, 2016; Kooyers et al., 2017; Moreira et al., 2018). Due to logistical and/or statistical complexity, the effects of more than one or two abiotic/biotic variables are not often considered simultaneously. Thus, while assessing the relative influence of multiple, simultaneous factors on defense trait production is critical to understand the evolution and ecology of defense trait similarity or variation across a landscape, we have very little empirical data to this end (Hunter, 2016).

Multiple studies across latitudinal and elevational gradients have shown the effects of climate and/or herbivory pressure on plant defense (Abdala‐Roberts et al., 2018; Carmona et al., 2020; Valdés‐Correcher et al., 2021; Vázquez‐González et al., 2019; Woods et al., 2012). Climate has known effects on herbivore species richness and abundance, as well as plant growth and life history strategy, all factors that can influence plant investment in defense (Bont et al., 2020; Kambach et al., 2016). Likewise, recent work demonstrates that the local environment can have a stronger effect on plant defenses than large‐scale macroclimatic clines (Sanczuk et al., 2020). Neighboring vegetative community composition may influence plant susceptibility or resistance to herbivores. Associational susceptibility and resistance lead to increases or decreases, respectively, in plant susceptibility to herbivory (Agrawal et al., 2006; Barbosa et al., 2009; Tahvanainen & Root, 1972). Associational susceptibility and resistance can be through indirect abiotic mechanisms, such as when neighboring plants alter resource availability to focal plants. Associational effects can also be more direct, through effects on herbivore and predator behavior or survival, through neighboring plants affecting herbivore movement to focal plants, or through the effects of neighbors on focal plant traits (e.g., Tagawa & Watanabe, 2021). The effects of neighboring plants may be at least partially dependent on climate (e.g., mean annual temperature; Poeydebat et al., 2020), as well as local resources and past ecological histories (Rotter & Rebertus, 2015). Thus overall, we expect climate, resource availability, herbivore pressure, and associated herbivore and vegetative communities to jointly shape phytochemical evolution.

The co‐evolutionary relationship between herbivores and plants is well documented (e.g., Becerra, 1997; Ehrlich & Raven, 1964; Janz, 2011). Reciprocal interactions exist between herbivore community composition and defense traits. Secondary chemistry affects herbivore community structure (e.g., Barker et al., 2018; Forkner et al., 2004; Glassmire et al., 2016; Wimp et al., 2007). Likewise, herbivores exert selection on physical and chemical defense traits (Mauricio & Rausher, 1997; Rausher & Simms, 1989; Shonle & Bergelson, 2000). Diversity of phytochemical defenses is associated with diversity of the herbivore community and amount of herbivory (Richards et al., 2015; Salazar et al., 2018). If particular herbivores or feeding guilds exert consistent selection pressures on multiple related plant populations, we might expect resistance arsenals (the type and variation of phytochemical resistance traits) in those populations to respond in similar ways or to converge. Alternatively, if abiotic drivers act more strongly in shaping phytochemical differences across plant populations, we might expect the effect of herbivore community similarity to be less apparent.

In this study, we explore the biogeographic structure of plant phytochemical resistance arsenals and the relative influences of factors that might contribute to them, in populations of yellow monkeyflower (*Erythranthe guttata* (DC.) G.L. Nesom; synonym: *Mimulus guttatus* DC.) across its native and introduced range. Specifically, we (1) use a greenhouse common garden to characterize genetic‐based variation in phytochemical resistance via phenylpropanoid glycoside (PPG; Holeski et al., 2013) concentrations in 41 populations of yellow monkeyflower from across six biogeographic regions in North America and the United Kingdom. We first determine the extent to which individual PPG variation is structured by geography relative to the genetic variation present within maternal families and next characterize the overall composition of PPG phytochemical resistance arsenals. We then ask (2) if physical distance, herbivore community similarity, vegetation community similarity, and/or several aspects of source climate similarity (temperature, precipitation, and seasonality) are correlated with phytochemical resistance arsenal differentiation. Lastly, we determine (3) which potential drivers contribute most strongly to the differentiation of phytochemical resistance arsenals among populations of yellow monkeyflower. Overall, we hope to better understand how abiotic conditions and biotic interactions shape plant defense traits.

## 2 MATERIALS AND METHODS

### 2.1 Study system

Yellow monkeyflower (*Erythranthe guttata* (DC.) G.L. Nesom; synonym: *Mimulus guttatus* DC.) is a model species in evolution, ecology, and genetics (Ritland & Ritland, 1996; Troth et al., 2018; Vickery, 1978; Wu et al., 2008). Populations occupy areas of perennial and ephemeral water from northern Mexico to central Alaska and from sea level to 3000 m in elevation. The species possesses tremendous morphological variation, with genetic‐based differences in life history and levels of defense, including constitutive and induced phenylpropanoid glycosides (PPGs) (Friedman et al., 2015; Holeski, 2007; Holeski et al., 2013; Kooyers et al., 2017; Lowry et al., 2008).

### 2.2 Field sampling

**FIGURE 1:**
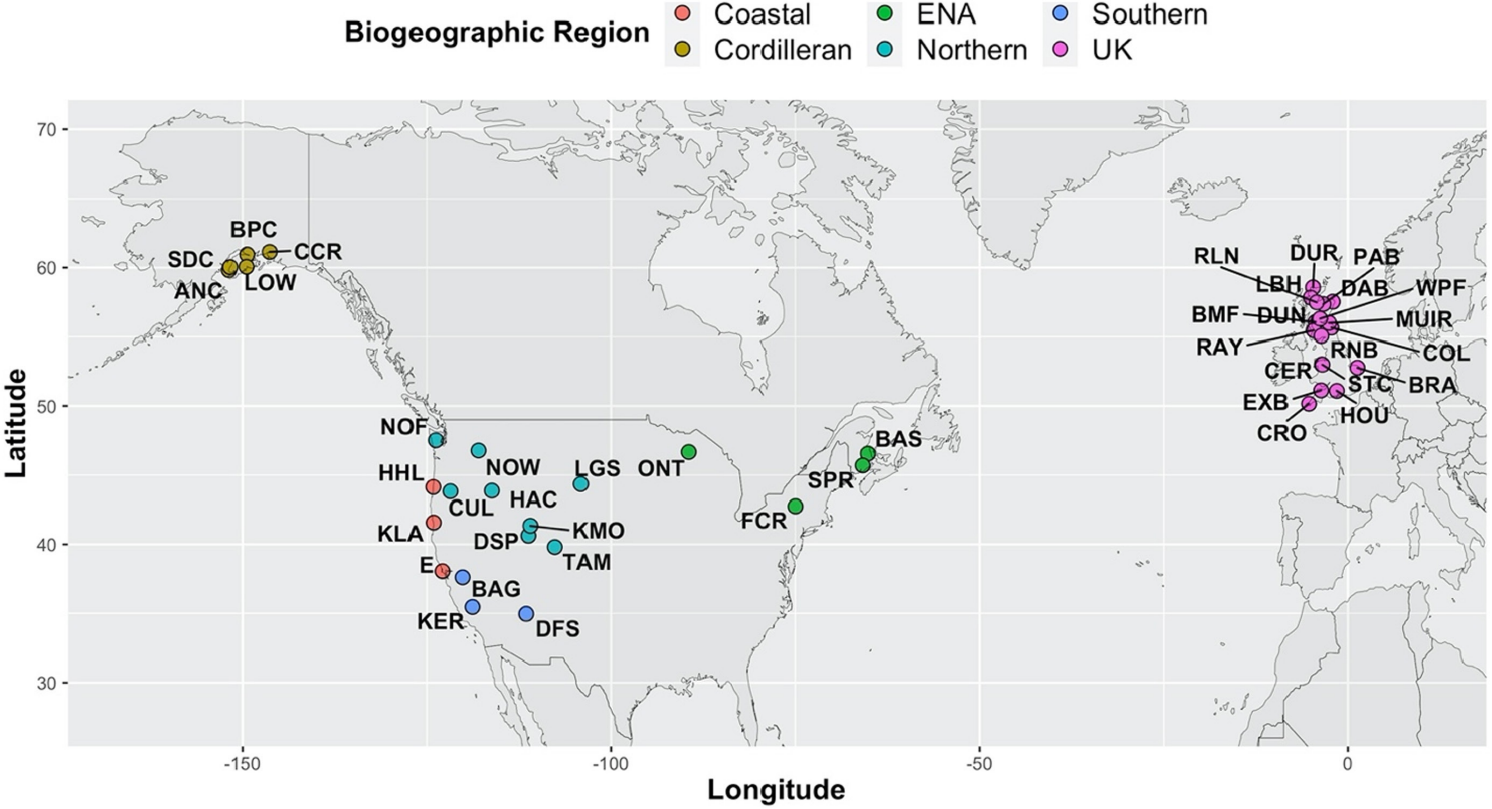
Map of the sampling locations of 41 yellow monkeyflower populations from six distinctive biogeographic regions. Biogeographic regions follow clade distinctions for Western North America (Twyford & Friedman, 2015) and the United Kingdom (Vallejo‐Marín et al., 2021), whereas introduced populations in Eastern North America represent multiple invasions and are thus paraphyletic (Vallejo‐Marín et al., 2021).

We sampled 41 yellow monkeyflower populations spanning the western hemisphere and comprising six distinct biogeographic regions (Figure 1). We sampled native populations from the four biogeographic clades of yellow monkeyflower in western North America (Twyford & Friedman, 2015; Twyford et al., 2020), including populations from the Coastal region (*n* = 3), the Cordilleran region (*n* = 5), the Northern region (*n* = 8), and the Southern region (*n* = 3). Yellow monkeyflower is native to western North America and was introduced into the United Kingdom (UK) approximately 200 years ago and subsequently reintroduced into Eastern North America (ENA) initially from UK populations and then subsequently through secondary invasions from other source populations (Vallejo‐Marín et al., 2021). We also sampled introduced populations from Eastern North America (*n* = 4) and from the United Kingdom (*n* = 18) (Figure 1). In total, we sampled 19 populations from the native range and 22 populations from the introduced range in the United Kingdom and Eastern North America (Figure 1). The closest population pair in our dataset was 5.9 km apart (within the UK), while the furthest population pair spanned 8650 km of physical distance (from the United Kingdom to Alaska).

For each of the 41 yellow monkeyflower populations, we documented the number of species present from each particular family of herbivores in the field as a measure of species richness. We considered a population of yellow monkeyflower to be a distinct group of closely growing plants that were likely interbreeding. Insects were classified at the family level while vertebrates and gastropods were assigned their own groups. Insect diet breadth is often predictable at the family level (Futuyma & Agrawal, 2009), and family‐level classification within insects has been used in a variety of evolutionary (Berenbaum et al., 1986; Descombes et al., 2017) and ecological studies (Bellamy et al., 2018; Godoy et al., 2019) as a reliable level of taxonomic resolution. Classification of vertebrate and gastropod damage was based on visual evidence of feeding damage, yellow monkeyflower site knowledge, and clear visual reliability of feeding evidence. We assessed herbivores using visual and sweep net surveys. Within the visual surveys, we searched for signs of feeding damage on the plants as well as presence of herbivores themselves. We spent 5 min searching in a 1 × 1 m patch of yellow monkeyflower and then expanded out systematically to the entire patch. Invertebrates were considered to be feeding on yellow monkeyflower if we directly observed feeding or if the invertebrates were on a plant and were a species that would likely consume yellow monkeyflower (i.e., the insect was an herbivore). When possible, for the latter case, we collected the animal alive and confirmed yellow monkeyflower consumption in the lab. Sweep netting was done only in areas that had high densities of yellow monkeyflower (90% or more) so as not to catch insects that may have been feeding on closely growing plants. Sweeps were completed after the visual surveys and timed in a similar manner. Herbivore surveys were conducted over 2 or more years, at varying points within the growing season (at least one late and one early in the growing season based on local conditions; Rotter et al., 2019).

We surveyed yellow monkeyflower‐adjacent vegetation communities by documenting the presence of all co‐occurring plant species at the family level. As with herbivores, we documented species richness as the number of species present from each particular family. Plants were considered to occur in the same community as yellow monkeyflower if they were physically very close (<1 m from a yellow monkeyflower plant), and/or likely directly competing with yellow monkeyflower for resources (e.g., for space or light, root competition, nutrient uptake, etc.). We used family‐level taxonomy for vascular plants as it may be a good predictor of herbivore specialization (Winkler & Mitter, 2008); this method also allows for a shared general approach of taxa across a worldwide biogeographic study (Guo et al., 1998; Qian, 1999). Our family‐level taxonomy follows that of APG IV (Angiosperm Phylogeny Group, 2016).

Finally, we collected seeds from each yellow monkeyflower population to use in a common garden experiment. We used this common garden approach as we were interested in only the genetic‐based traits of the populations. Although PPG concentrations can be plastic (Holeski et al., 2013), they have a strong genetic basis and broad patterns of PPG production are similar across environments (M. L. Blanchard, L. M. Holeski, unpublished data). In each population, we collected seeds from multiple flowers from each of >20 plants that were separated by at least a meter. We then grew seeds in the Northern Arizona University greenhouse for at least one generation to minimize possible maternal effects. Plants used were bred into multiple outcrossed maternal families, with crosses performed within each population. All plants were grown in a common environment for 6 months and rotated weekly. Plants were grown in Fafard 3B mix potting soil under 16 h high‐pressure sodium light and bottom flood watering with weekly fertilizer of 10‐30‐20 (Peters Professional Fertilizer).

### 2.3 Common garden defense trait measurements

We quantified chemical traits associated with yellow monkeyflower defensive arsenals in plants grown in a greenhouse common garden. We assayed the foliar concentrations of seven phenylpropanoid glycosides (PPGs), the predominant bioactive secondary compounds in the species (Holeski et al., 2013; Keefover‐Ring et al., 2014). These seven PPGs represent all PPGs that were detected in the samples. One leaf from the third true leaf pair was cut at the base of the petiole with scissors and flash frozen in liquid nitrogen before being transferred to a −20°C freezer. Tissue was then lyophilized using a pre‐chilled FreeZone triad freeze dry system (Labconco). Samples were stored, ground, and extracted as described in Rotter et al. (2018). On average, we sampled three individuals from each of four maternal families per population (*n* = 12 individuals per population) from each of the 41 yellow monkeyflower populations (*n* = 483 individuals in total). We quantified the PPG content of each sample via high‐performance liquid chromatography (HPLC; Agilent 1260 HPLC with a diode array detector and Poroshell 120 EC‐C18 analytical column [4.6 × 250 mm, 2.7 μm particle size]; Agilent Technologies) maintained at 30°C, as described in Kooyers et al. (2017). Seven PPGs were found: unknown PPG 10, calceolarioside A, calceolarioside B, conandroside, verbascoside, mimuloside, and unknown PPG 16 (Keefover‐Ring et al., 2014).

### 2.4 Statistical analysis

#### 2.4.1 Modeling PPG concentrations and variance partitioning analysis

We used linear mixed models (*lmer* function from the *lme4* package, Bates et al., 2015) and generalized linear mixed models (*glmer.nb* function from *lme4*) in R (R Core Team) to model the concentrations of each of the seven PPGs, fitting fixed effects for biogeographic region and population, and a random effect for maternal line (specific model specifications are provided in Table S1). Concentration data were overdispersed for all PPGs, with many low values and a decreasing number of samples with higher concentrations. Of the 3381 total PPG measurements (seven PPG compounds for each of 483 plants), 56 individual measurements (1.66%) had values of zero. To facilitate downstream regression analysis, we added scalar values to all PPG data, based on the lowest measured non‐zero concentration (0.0003–0.043) for that PPG, and then log‐transformed PPG concentrations. We elected to use transformations, when possible, instead of fitting models using alternative distributional families, to facilitate variance partitioning analysis (see below). We diagnosed model performance using the *simulateResiduals* function from the *DHARMa* package (Hartig, 2021), requiring congruence of observed and expected residuals as assessed through Q‐Q plots and via the *testDispersion* function. We modeled PPGs that were inadequately modeled using log‐transformed concentration data (calceolarioside A and conandroside) using negative binomial GLMMs with non‐transformed raw data (Table S1).

We conducted variance partitioning analysis using the *partR2* (Stoffel et al., 2021) and *rptR* (Stoffel et al., 2017) packages in R. For each PPG model, we decomposed total variance into semi‐partial *R* ^2^ indicating the proportion of total variance explained by the fixed effects (i.e., marginal *R* ^2^) of biogeographic region and population (fit individually, and also jointly, thus representing the full effect of geography), as well as the proportion of variance explained by genotypic variation at the level of maternal family. We passed the linear mixed models described above (the *partR2* and *rptR* packages do not currently accept negative binomial model families, so we were unable to partition variance for calceolarioside A and conandroside) to the *partR2* function (with 1000 bootstrap replicates) to determine 95% confidence intervals for marginal *R* ^2^ for both region and population, as well as the joint effect of these co‐varying geographic predictors. Next, we fit the same models again using the *rpt* function (with 1000 bootstrap replicates) to determine the proportion of variance explained by maternal family.

#### 2.4.2 Characterizing the composition of PPG phytochemical resistance arsenals, herbivore and vegetation communities, and climates among biogeographic clusters of yellow monkeyflower

For each of the seven PPGs, we used the models described above to estimate least‐square means for each population using the *emmeans* function from the *emmeans* package (Lenth, 2021). We back‐transformed estimates that were generated using log‐transformed data and subtracted the scalar values to correct model predictions as needed. Using these population‐level estimates, we conducted Nonmetric Multidimensional Scaling (NMDS) using the *metaMDS* function from the *vegan* package (Oksanen et al., 2020) to visualize how populations and biogeographic clusters segregated in multi‐dimensional phytochemical arsenal space. We added convex hulls to visually demarcate biogeographic regions using the *ordihull* function in *vegan*.

We used the *vegdist* (with Bray‐Curtis distance) and *betadisper* functions from *vegan* to test for homogeneity of variances among biogeographic regions. A finding of no evidence for differences in group dispersion confirms that downstream differences assessed through permutational multivariate analysis of variance reflect differences in group means, as opposed to group variances. We used PERMANOVA (*adonis2* function in *vegan*) to test for differences in multi‐dimensional phytochemical arsenal space among biogeographic clusters. Next, we used pairwise PERMANOVA (*adonis.pairs* function from the *EcolUtils* package, Salazar, 2021) to assess differences in arsenal composition among individual pairs of biogeographic regions.

We followed the same procedures to visualize and assess differences in the composition of the herbivore and vegetation communities across the six biogeographic clusters of *M. guttatus*. We first created a matrix describing the herbivore community composition at each of the 41 populations based on 20 arthropod families, and gastropods and mammals, and then created a similar vegetation community matrix based on the species richness of plant neighbors from 50 plant families. Thus, NMDS and PERMANOVAs for herbivores and vegetation communities were based on our field observations, whereas similar analyses for the PPGs were based on models generated from trait data collected under shared environmental conditions in the greenhouse.

To assess climatic differences across biogeographic regions, we first extracted data for 19 bioclimatic variables reflecting present‐day conditions for each population. We extracted climatic data from the *WorldClim* global climate database (Fick & Hijmans, 2017) at 2.5 min of a degree resolution, using the *getData* function from the *raster* package (Hijmans, 2021) in R (R Core Team). Bioclimatic variables included measured variables such as mean annual temperature and precipitation, as well as derived variables such as mean diurnal temperature range, and temperature and precipitation seasonality (Fick & Hijmans, 2017). We characterized the overall climate based on all 19 *bioclim* variables using the same procedures as above. We also characterized climatic similarity based on three dimensions of the climate – temperature (bio1, bio5, bio6, bio8, bio9, bio10, bio11), precipitation (bio12, bio13, bio14, bio16, bio17, bio18, bio19), and seasonality (bio2, bio3, bio4, bio7, bio15). Since we calculate climatic distance between populations using Bray–Curtis distance, we added a scalar value to all bioclimatic variables (bio1, bio6, bio8, bio9, bio11) that contained negative values, bringing all values for that variable into a positive range.

#### 2.4.3 Assessing relationships between phytochemical arsenal similarity and potential biotic and abiotic predictors

We first calculated community dissimilarities among all populations with respect to phytochemical defensive arsenals, herbivore and vegetation communities, and temperature‐based, precipitation‐based, and seasonality‐based measures of the climate using the *vegdist* function (with Bray–Curtis dissimilarity) from the *vegan* package. We tested for associations between phytochemical arsenal similarity and potential correlates using Mantel tests (*mantel* function from *vegan*) based on dissimilarity matrices. Next, to assess relationships between geographic distance and each of our metrics of community similarity (phytochemical arsenals, herbivores, vegetation, and the three aspects of climate), we calculated pairwise physical distances among all 41 yellow monkeyflower populations based on their latitude and longitude positions using the *pointDistance* function from the *raster* package (Hijmans & Van Etten, 2020), which calculates the shortest distance between two points (i.e., “great‐circle” distances). We again used Mantel tests to assess the relationship between physical distance and phytochemical arsenal distance, as well as that between physical distance and each of the five potential correlates of phytochemical differentiation.

#### 2.4.4 Determining the relative effects of potential drivers of phytochemical arsenal differentiation

Due to pervasiveness of isolation by distance, in which community differences increase with the physical distance between populations (Orsini et al., 2013; Wright, 1943), we are often interested in jointly assessing the relative strengths of both geographic and ecological drivers on community dissimilarity, as opposed to simply controlling for geographic proximity. To address this challenge, we implemented an extension of BEDASSLE (*Bayesian Estimation of Differentiation in Alleles by Spatial Structure and Local Ecology*), a method originally designed to quantify the relative contributions of geography and ecology on genetic differentiation between individuals or populations (Bradburd, 2013; Bradburd et al., 2013). BEDASSLE models a response (i.e., allele frequencies in a group of populations) in which the covariance structure decreases with both physical and ecological distances separating populations and uses a Markov chain Monte Carlo (MCMC) algorithm to estimate effect sizes of model parameters. Here, BEDASSLE generated standardized effect sizes for ecological predictors (aE), indicating how a one‐unit change in each predictor contributes to differentiation in phytochemical arsenal similarity. We utilized BEDASSLE to infer the relative contributions of physical distance and five measures of ecological similarity (herbivores, neighboring vegetation, and temperature‐, precipitation‐, and seasonality‐based measures of the climate) on the differentiation of phytochemical resistance arsenals. A similar extension of BEDASSLE has recently been employed to assess the relative importance of factors contributing to microbiome community composition in baboons (Grieneisen et al., 2019). Additional details on BEDASSLE methods can be found in Appendix S1.

## 3 RESULTS

### 3.1 Variation in phenylpropanoid glycoside (PPG) production

**FIGURE 2:**
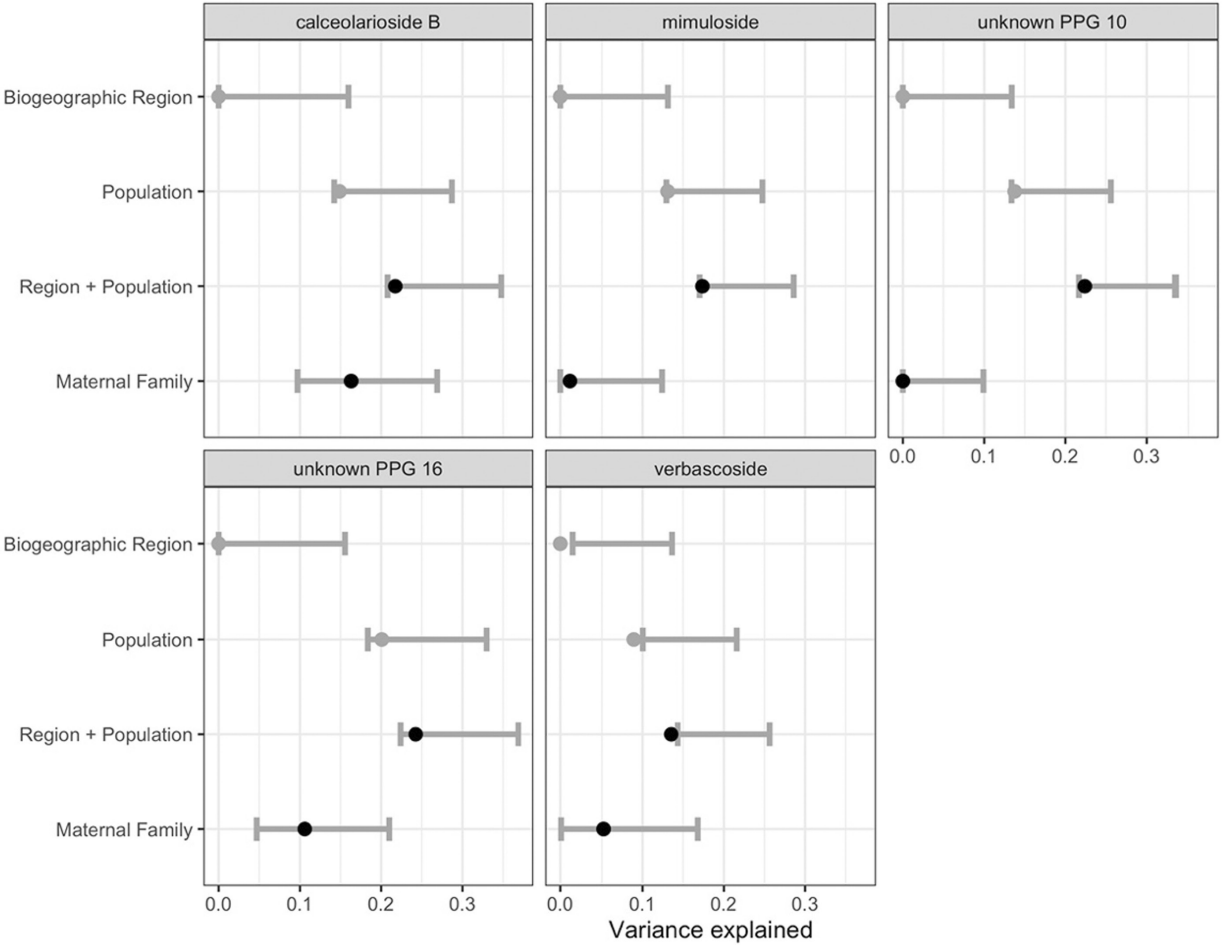
Semi‐partial *R* ^2^ indicating the proportion of variance in phenylpropanoid glycoside (PPG) production explained by the geographic effects of biogeographic region and population, and the within‐population effects of maternal family. Gray error bars represent 95% confidence intervals for marginal *R* ^2^ estimates; black dots represent point estimates for geographic variation (the joint effects of region + population) and within‐population genetic variation (maternal gamily); gray dots represent the effects of region and population when assessed individually.

Under shared environmental conditions in the greenhouse common garden, we observed substantial variation in PPG concentrations. Individual plants varied by factors of up to ~1700×–3600× for most PPGs (and up to 14,000× for calceolarioside B), and on average, populations varied by factors of 5×–21× (with calceolarioside B varying by a factor of 48× among some populations). For all five of the seven PPGs for which we were able to decompose variance, the joint effect of biogeographic region and population explained a much greater portion of variance in PPG production than did maternal family (Figure 2, Figure S1). On average, the effect of geography (composed of the joint effect of biogeographic region and population) explained 19.9% of the variation in genetically based PPG production compared with 6.7% explained by the genetic effects of maternal family (Figure 2).

### 3.2 Compositional similarity among biogeographic clusters of *M. guttatus*

#### 3.2.1 Phytochemical resistance arsenals

**FIGURE 3:**
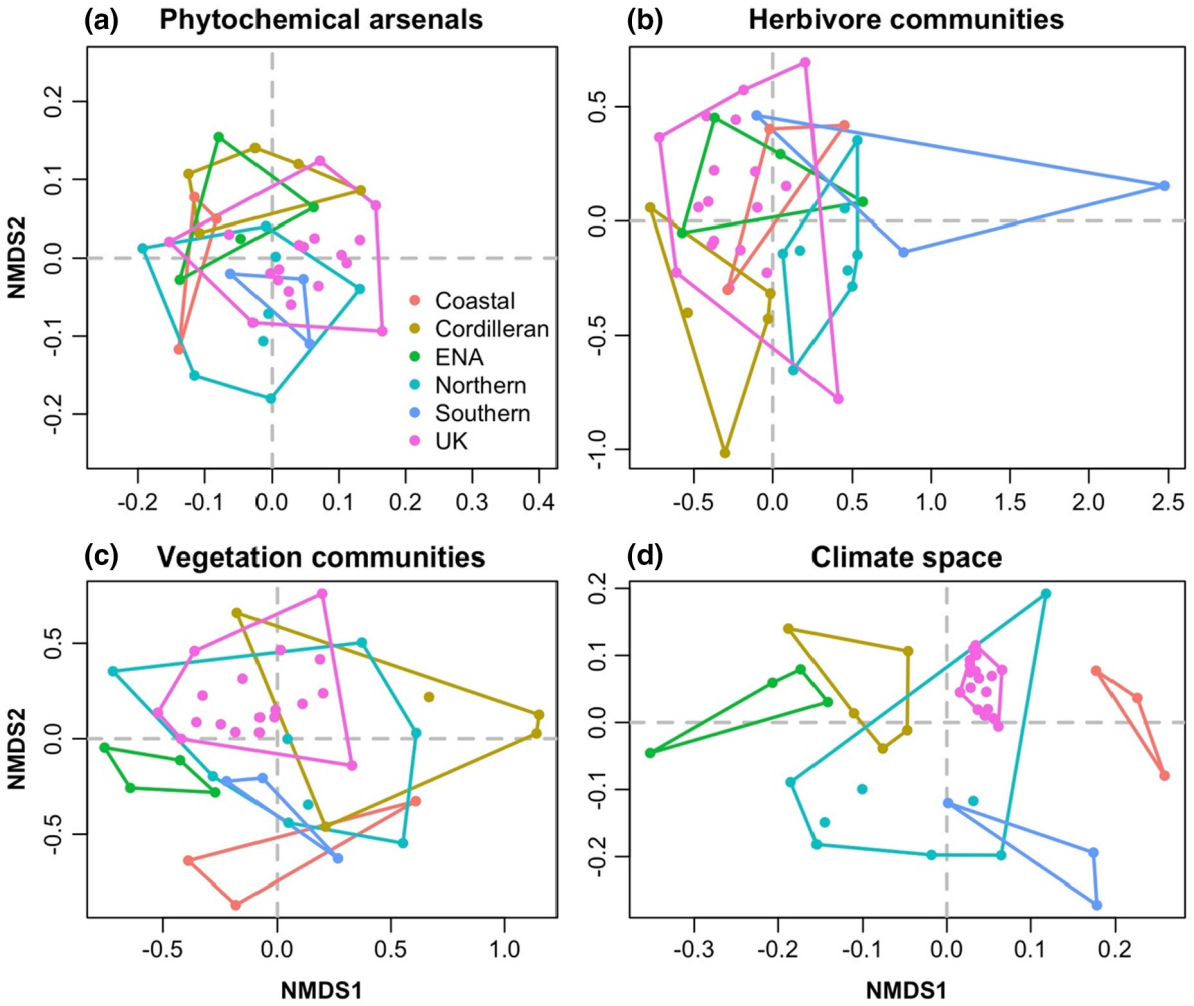
Non‐metric multi‐dimensional scaling (NMDS) plots depicting the compositional similarity of phytochemical resistance arsenals, herbivore and vegetation communities, and overall climates of 41 populations of yellow monkeyflower from six different biogeographic regions. (a) Phytochemical resistance arsenals based on the concentrations of PPGs as measured in plants grown in a greenhouse common garden; (b) Herbivore composition based on the species richness of arthropods, gastropods, and mammal herbivores as observed in the field; (c) Vegetation composition based on the species richness of plant neighbors as observed in the field; and (d) Climate space based on 19 bioclimatic variables extracted from WorldClim.

Despite some overlap in multi‐dimensional phytochemical resistance trait space among yellow monkeyflower populations from different biogeographic regions (Figure 3a; non‐metric *R* ^2^ = .96; NMDS stress = 0.20), there was a significant difference in arsenal composition among regions (PERMANOVA *p* = .04, *F*_5,40_ = 2.16, *R* ^2^ = .24). This difference reflected significant (*p* < .05) and trending (*p* < .10) pairwise differences among the UK and Cordilleran and other biogeographic regions (Table S2) rather than differences in compositional dispersion among biogeographic clusters (*p* = .67, *F*_5,40_ = 0.64).

#### 3.2.2 Herbivore communities

Several biogeographic regions had relatively distinctive herbivore communities (Figure 3b; non‐metric *R* ^2^ = .97; NMDS stress = 0.18), and there was a significant difference in herbivore composition among regions (PERMANOVA *p* < .001, *F*_5,40_ = 2.53, *R* ^2^ = .27). Significant pairwise differences were again driven by the UK and Cordilleran clusters (Table S3). Biogeographic clusters did not show differences in group dispersion with respect to the composition of their herbivore communities (*p* = .31, *F*_5,40_ = 1.25).

#### 3.2.3 Vegetation communities

The compositions of vegetation communities across biogeographic clusters were strongly differentiated (Figure 3c; non‐metric *R* ^2^ = .94; NMDS stress = 0.24). This differentiation reflected mean differences in vegetation composition (PERMANOVA *p* < .001, *F*_5,40_ = 3.28, *R* ^2^ = .32) rather than differences in group dispersion (*p* = .52, *F*_5,40_ = 0.86). The UK cluster showed significant differences between all other biogeographic regions (Table S4), and the vegetative composition of the Cordilleran region was again significantly different from most other regions (Table S4).

#### 3.2.4 Climate

Populations from each biogeographic region (Figure 1) were strongly clustered in multivariate climate space (Figure 3d; NMDS non‐metric *R* ^2^ = .99; NMDS stress = 0.10). Overall, there were significant differences in group means (PERMANOVA *p* < .001, *F*_5,40_ = 23.35, *R* ^2^ = .77) with no differences in group dispersion (*p* = .55, *F*_5,40_ = 0.83). Here, almost all pairwise comparisons among biogeographic regions were significantly different (Table S5).

### 3.3 Correlations between phytochemical arsenal similarity and potential biotic and abiotic predictors

**FIGURE 4:**
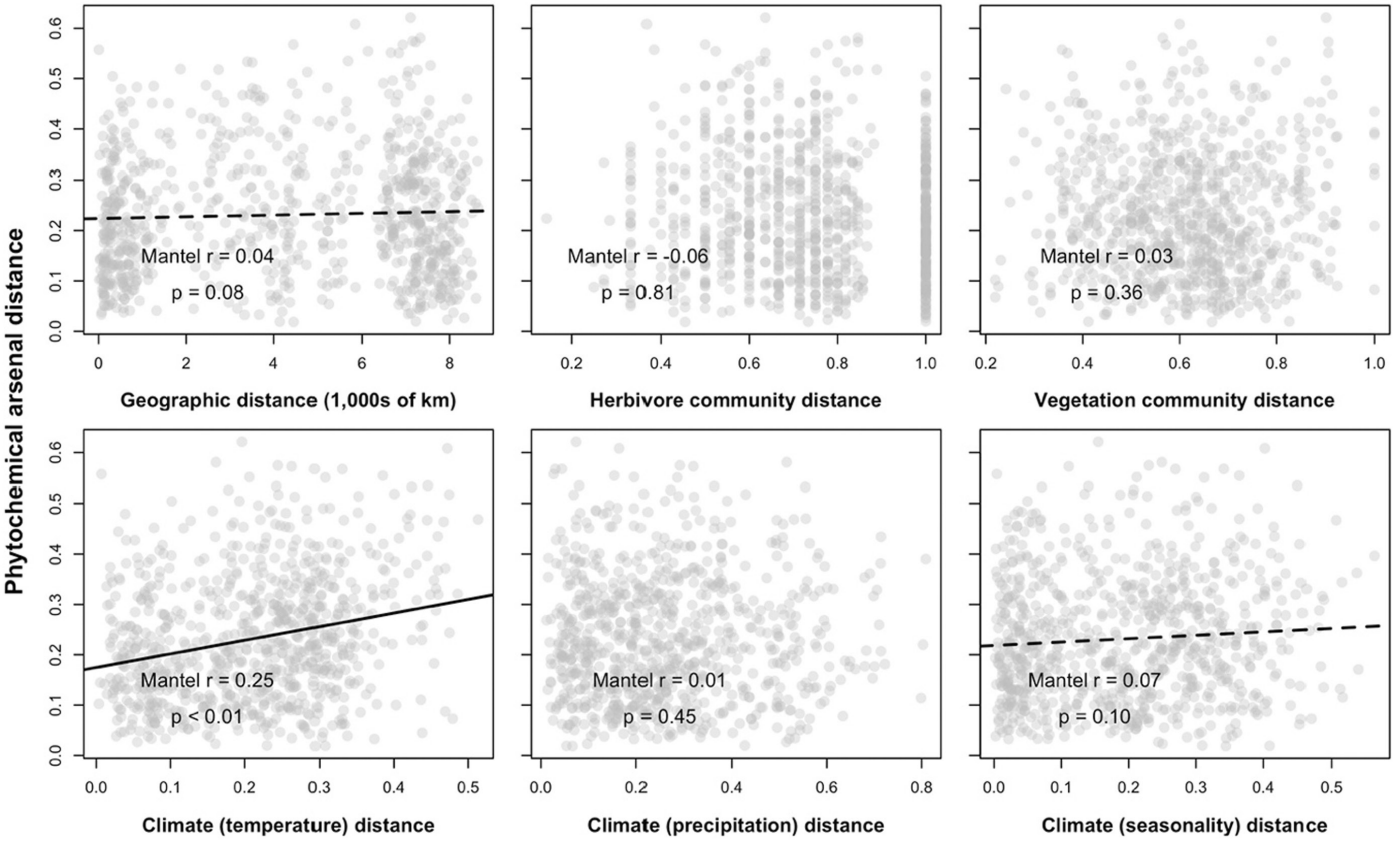
Associations between physical distance, two potential biotic correlates (herbivore and vegetation community composition), and three potential abiotic correlates (temperature‐, precipitation‐, and seasonality‐based components of climatic similarity) of phytochemical arsenal differentiation. Solid lines indicate statistically significant associations (*p* < .05), dashed lines indicate trending associations (*p* < .10).

We found some evidence for a positive association between phytochemical arsenal dissimilarity and the physical distance separating yellow monkeyflower populations (Figure 4a; Mantel *r* = .04, *p* = .08), indicating that populations further from one another tended to be differentiated in their arsenal composition. There was no relationship between herbivore community differences and phytochemical arsenal composition (Figure 4b; Mantel *r* = −.06, *p* = .81), nor between vegetation community differences and arsenal composition (Figure 4c; Mantel *r* = .03, *p* = .36). Among the three components of the climate we assessed, differences in temperature‐based components of the climate were significantly positively correlated with phytochemical arsenal differentiation (Figure 4d; Mantel *r* = .25, *p* < .01), there was no relationship with precipitation‐based climate similarity (Figure 4e; Mantel *r* = .01, *p* = .45) and a trend toward a positive association between differences in climate seasonality and phytochemical arsenal composition (Figure 4f; Mantel *r* = .07, *p* = .10). Additionally, we observed spatial autocorrelation in all potential predictors, as community or multi‐dimensional similarities for all five potential predictors of phytochemical arsenal differentiation were highly significantly correlated with the physical distance separating populations (Figure S2).

### 3.4 Determining the relative effects of potential drivers of phytochemical arsenal differentiation

**FIGURE 5:**
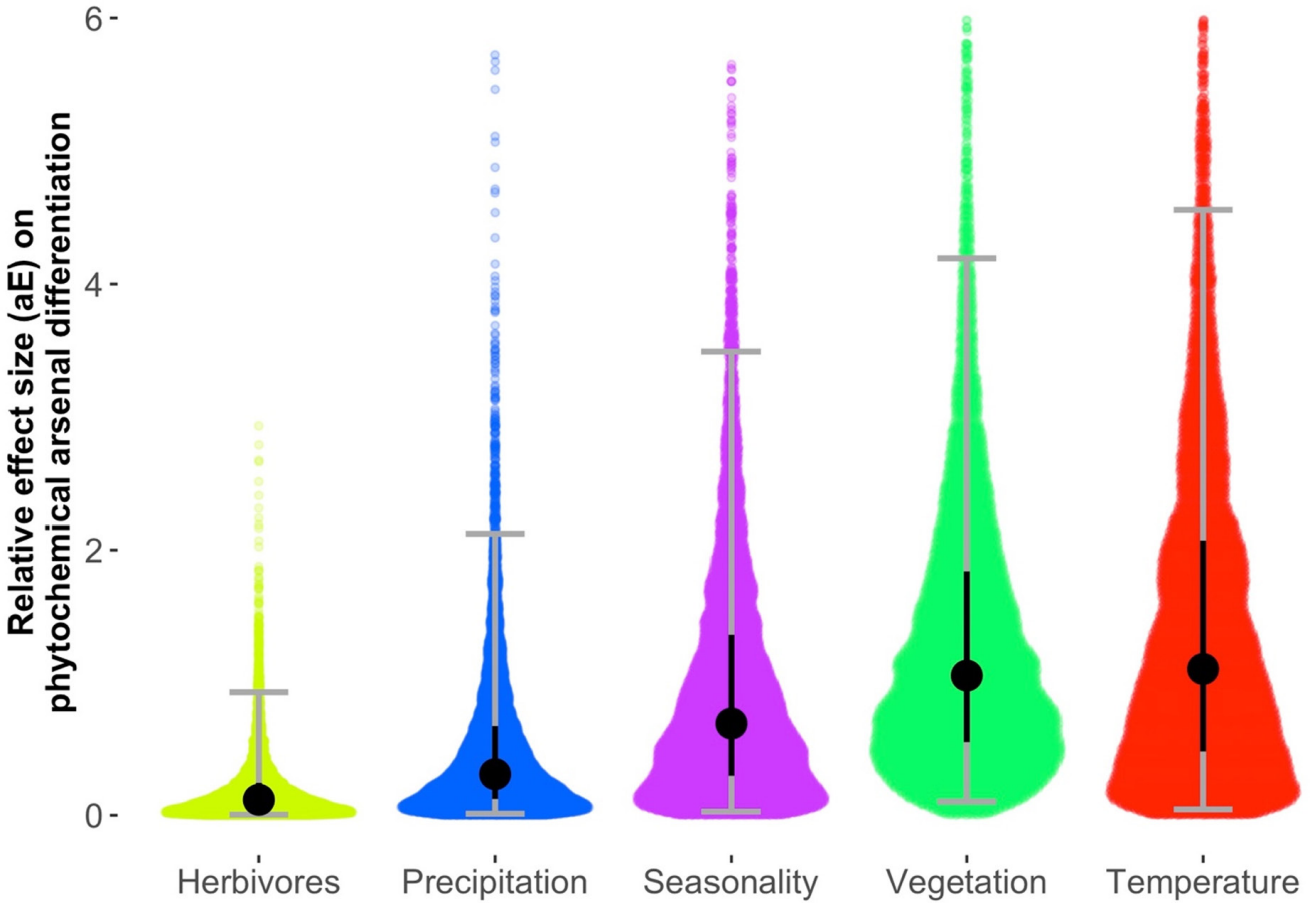
Density plots of effect sizes (aE) of biotic and abiotic contributors to the differentiation of phytochemical resistance arsenals among populations of yellow monkey flower. Colored points represent individual post burn‐in draws from a consensus posterior distribution, sampled every 10,000 generations from 10 separate, 10‐million generation MCMC sampling algorithms conducted in BEDASSLE. Large points represent medians from the consensus post burn‐in posterior distribution, vertical black bars depict the interquartile ranges, and gray error bars show 95% credible intervals.

The BEDASSLE analysis provided standardized effect sizes (aE), indicating how a one‐unit change in each of five ecological predictor variables would contribute to differentiation in phytochemical arsenal similarity. A Bray–Curtis distance of one reflects complete differences in community composition; thus, effect sizes can be interpreted as how complete turnover in one of the predictor variables would affect arsenal differentiation. Differences in the herbivore communities (median aE = 0.12) and precipitation regimes (median aE = 0.31) had the smallest effects on phytochemical arsenal differentiation; climate seasonality had an intermediate effect (median aE = 0.69); whereas vegetation communities (median aE = 1.06) and temperature regimes (median aE = 1.12) were the strongest potential drivers of arsenal differentiation across populations (Figure 5).

Bayesian estimation of differentiation in alleles by spatial structure and local ecology also provides an effect size for physical distance (aD); here, 1000‐km of physical distance between populations contributes to a median 0.17‐unit change in phytochemical arsenal dissimilarity. For the sake of comparison, the effect of complete turnover in herbivore communities is roughly equivalent to moving 700 km of physical distance in its effects of arsenal differentiation, whereas a completely different precipitation regime would result in about twice that effect on arsenal differentiation (aE = 0.31 vs. aD = 0.17). Total shifts in vegetation communities or temperature regimes would result in about 6× larger effects on phytochemical arsenal differentiation compared with moving 1000 km.

## 4 DISCUSSION

Plant resistance traits evolve according to past abiotic and biotic environmental factors and influence current ecological interactions at multiple trophic levels. The characterization of drivers of genetic‐based resistance trait variation across a landscape can provide insight into the ecological factors that have shaped the evolution of these patterns, as well as allow for predictions for future ecological interactions. We assessed the relative influences of abiotic and biotic drivers of genetic‐based defense trait variation between plant populations in a study of 41 yellow monkeyflower populations across western and eastern North America and the United Kingdom. Our study combines observational field data and common garden‐based trait measurements of phytochemical resistance traits. Most previous work on the evolution and differentiation of resistance arsenals has focused on single drivers. We jointly assess the relative strengths of both geographic and multiple ecological variables on community dissimilarity, rather than simply controlling for geographic proximity. This approach allows us to better understand the complex ways in which multiple co‐varying forces may contribute to phytochemical and resistance trait differentiation in natural populations.

We find that biogeographic groups of yellow monkeyflower populations inferred from genetic data (Twyford & Friedman, 2015; Twyford et al., 2020) experience significantly different climates, different compositions of herbivore communities, co‐occur with different vegetative communities, and have distinct phytochemical resistance arsenals. Similarities in climate as well as herbivore and vegetative communities decline with increasing physical distance separating yellow monkeyflower populations. A weak association between physical distance and plant resistance arsenal composition may indicate that the climate, herbivores, and plant neighbors have countervailing forces on plant defensive arsenals, causing them to converge under some circumstances. Physiological or genetic constraints could also contribute to this pattern, if there is a limited subset of defensive trait space that yellow monkeyflower populations are able to occupy.

### 4.1 The interaction between climate and genetic‐based trade‐offs for defenses

Previous studies of defense arsenals in yellow monkeyflower focusing on phytochemical defense have not found strong genetic constraints among defense traits (Holeski et al., 2014; Kooyers et al., 2020). However, genetic correlations between defense and life history strategy may play some role in the climate‐related patterns that we observe in this study. Genetic‐based trade‐offs between levels of phytochemical defense and traits related to key life history traits (e.g., time to first flower, allocation to vegetative biomass vs. reproductive traits) have been shown in both annual (Kooyers et al., 2017) and annual/perennial systems (Lowry et al., 2019; Rotter et al., 2019). At least some of these correlations also have strong associations with climate; for example, in annual monkeyflower populations, the length of the growing season (defined by both precipitation and temperature) is positively correlated with concentrations of phytochemical defense (Kooyers et al., 2017). This intra‐specific pattern is similar to macroevolutionary patterns across the monkeyflower phylogeny, whereby PPG concentrations are significantly influenced by mean annual temperature, amount of precipitation, and growing season length (Holeski et al., 2021). There are thus likely indirect effects of climate on monkeyflower resistance arsenals mediated through plant life history strategy.

The non‐native populations in the United Kingdom represent a somewhat distinctive portion of multi‐dimensional resistance trait space, relative to populations in North America. This is potentially related to unique multidimensional climate space and vegetation communities occupied by the UK populations relative to native populations (Figure 3). It could also arise from founder effects, unmeasured environmental variables, and/or the result of competitive interactions required for successful establishment (Rotter et al., 2019). In comparison, the non‐native populations in Eastern North America also exist in unique climate space and have differing associated vegetative communities, relative to the native North American populations, but do not always occupy unique multidimensional resistance trait space (Table S2). Previous work by Rotter et al. (2019) illustrated genetic‐based trade‐offs between resistance traits and plant fitness in the United Kingdom, but not in Eastern North America. It is possible that these allocational trade‐offs in combination with novel climatic variables or ecological forces related to competitive interactions might underlie the observed differences in resistance arsenals between the non‐native UK populations and Eastern North American populations.

### 4.2 The relevance of intra‐ and inter‐ specific variation in resistance traits

The spatial distribution of plant phytochemical variation across a landscape is both influenced by and influences trophic interactions and nutrient dynamics in an ecosystem (Hunter, 2016; Northup et al., 1998). Most studies of the effects of functional traits on community and ecosystem functioning have historically focused on species mean trait values, rather than incorporating intra‐species variation (Siefert et al., 2015). More recently, the relevance of intra‐species variation for community assembly and stability and ecosystem processes have been highlighted (Albert, Thuiller, Yoccoz, Douzet, et al., 2010; Albert, Thuiller, Yoccoz, Soudant, et al., 2010; Bolnick et al., 2011; Crutsinger et al., 2006).

Identification of intra‐specific spatial patterns in functional traits such as plant defenses can be a first step in linking them to spatial patterns of nutrient availability (Hunter, 2016; Westerband et al., 2021). While we do not include analysis of soil in our study, doing so in future studies may provide insight into factors or processes that drive differences among populations in resource acquisition and allocation patterns. Here we find differences in phytochemical resistance arsenal composition among biogeographic regions (Figure 3a). Different genetic clades and biogeographic clusters of yellow monkeyflower may be resisting herbivory in somewhat different ways, i.e., via at least partially differentiated arsenal compositions. For example, while there is moderate overlap in the herbivore community multi‐dimensional space among some biogeographic clusters (Figure 3b), the UK biogeographic cluster occupies a portion of relatively unshared resistance trait space (Figure 2a). This highlights the importance of considering populations across a species range in efforts to understand the ecological and evolutionary processes underlying patterns of defense trait production.

While our study focuses on variation among populations of the same species, the results of our work are complementary to previous studies of inter‐species variation in resistance traits at community and landscape levels. These studies have been conducted in multiple plant systems/regions, and predictive variables include climate, topographic, and edaphic variables, as well as herbivore pressure. In one such inter‐specific study at a landscape level across a large number (400+) plant species, phytochemical richness across species could be predicted in part as a function of climatic factors and soil moisture (Defossez et al., 2021). Similarly, here at the intraspecific scale, climatic variation is associated with compositional differences in phytochemical resistance arsenals. Future work looking at the relative influence of drivers of inter‐ versus intra‐specific patterns might be used to strengthen inferences of common influences on resistance trait patterns at different scales across the landscape.

### 4.3 Relative effects of ecological predictors on resistance trait similarity—interpretations and important caveats

A diversity of biotic and abiotic drivers may simultaneously contribute to the evolution of plant defensive arsenals (Denno & McClure, 1983; Hunter, 2016), yet the relative contribution of individual drivers may be difficult to ascertain due to biological processes (e.g., constraint, convergence, and/or complexity) or methodological reasons. Another issue is likely in the precision and accuracy of available data. In our study, for example, the abiotic factors (i.e., climatic data) come from long‐term datasets and are likely substantially more precise and accurate than the herbivore data, which came from much more temporally limited sampling. The added noise from limited sampling and the use of taxa occurrence at the family level rather than a more fine‐scale parameter could be reducing our ability to detect biological signal. Limited sampling likely affected the herbivore data to a greater extent than the vegetation data, given the greater ephemerality of herbivores relative to neighboring plants. Given that other work in yellow monkeyflower shows that herbivore communities do impose selection on natural populations, with PPGs affecting the extent of herbivory (Scharnagl et al., 2022), we predict that a stronger herbivory signal would be found with more comprehensive sampling.

Abiotic and biotic factors may drive phytochemical resistance arsenal differences, and these potential predictors may often be correlated themselves. From a statistical standpoint, when modeling a response resulting from pairwise geographic distance and pairwise ecological distances, partial Mantel approaches may be inappropriate when potential predictors show spatial structure or autocorrelation (Guillot & Rousset, 2013; Legendre et al., 2015). Multiple matrix regression, which can model a community similarity response as a function of multiple pairwise ecological distance matrices, and thus produces an effect size for each predictor variable (Lichstein, 2006), would be a conceptually tractable solution for answering our question. Such an approach, however, can also be inappropriate for inferring the relative importance of multiple individual explanatory variables when the variables themselves are strongly correlated (Wang, 2013). Here for example, the makeup of herbivore or vegetation communities might depend on the physical proximity separating plant populations, while the compositions of the herbivore and vegetation communities themselves might also be correlated.

To address these challenges, we utilized BEDASSLE, a population genetic method originally designed to model F_st_ based on unlinked loci as a joint function of ecological similarity and physical distance (Bradburd et al., 2013). The flexibility of BEDASSLE allows it to address questions in population genetics as well as in community ecology; that said, interpretation of the results obtained from our non‐standard application requires careful consideration in light of the model assumptions (Appendix S2). Overall, this analysis suggests that the temperature is a relatively stronger driver of phytochemical arsenal differentiation compared with climatic seasonality and also a much stronger driver than localized precipitation regimes (Figure 5). We hypothesize that the effects of temperature are indirect, possibly filtered through effects of temperature on plant life history strategy.

## 5 CONCLUSIONS

Strengths of our study lie in our broad geographic sampling across the full range of yellow monkeyflower in the northern Hemisphere (Figure 1), our use of a common garden approach to assess genetic‐based variation in phytochemical arsenal composition across this range, and the use of novel statistical framework to jointly disentangle multiple abiotic and biotic factors that may affect plant phytochemical resistance traits. We find drastic differences in the relative strength of the effects that abiotic and biotic variables have on resistance arsenals. At least at the resolution of our available data, abiotic factors related to temperature and seasonality, and biotic factors associated with neighboring plant communities, play an outsized role in predicting resistance arsenal similarity compared with precipitation regimes or herbivore communities. Temperature and seasonality variables impose strong selection on many aspects of plant life history strategy; genetic correlations between life history and resistance traits may play a role in our observed patterns. A long‐standing goal of plant resistance trait evolution studies is to better understand the relative contributions of biotic and abiotic interactions. Our results are complementary to the large body of literature supporting the independent roles of climate, herbivore communities, and neighboring plant communities in shaping plant resistance traits. Disentangling the relative roles of abiotic/biotic factors in shaping patterns of intraspecific trait variation will allow greater insight into the past ecology of these populations, as well as their evolutionary trajectories. Future work based on detailed herbivore sampling is needed to better understand the relative effects of biotic and abiotic selection pressures in shaping phytochemical arsenals in plants.

## AUTHOR CONTRIBUTIONS

**Michael C. Rotter:** Conceptualization (supporting); funding acquisition (supporting); investigation (lead); writing – original draft (supporting); writing – review and editing (supporting). **Kyle Christie:** Conceptualization (equal); formal analysis (lead); writing – original draft (equal); writing – review and editing (supporting). **Liza M. Holeski:** Conceptualization (lead); funding acquisition (lead); investigation (supporting); writing – original draft (lead); writing – review and editing (lead).

## Supporting information


Appendix S1

## Data Availability

The data used in this study will be placed in a data repository such as Dryad after publication.
