# Supplementary material for: Climate and the biotic community structure plant resistance across biogeographic groups of yellow monkeyflower
Source: Ecol Evol. 2022 Nov 22;12(11):e9520. doi: 10.1002/ece3.9520 (PMC9682197; doi:10.1002/ece3.9520)
Supplement: Supplementary file 1 — Appendix S1 [file ECE3-12-e9520-s001.docx]

**SUPPLEMENTARY TABLES**

| **PPG** | **model type** | **raw or transformed response** | **model family** | **model specification** |
| --- | --- | --- | --- | --- |
| unknown PPG 10 | linear mixed model | log transformation | Gaussian | log(unknown PPG 10) concentration ~ Subregion + Population + (1\|Maternal Family) |
| calceolarioside A | generalized linear mixed model | raw data | Negative Binomial | calceolarioside A concentration ~ Subregion + Population + (1\|Family) |
| conandroside | generalized linear mixed model | raw data | Negative Binomial | conandroside concentration ~ Subregion + Population + (1\|Family) |
| verbascoside | linear mixed model | log transformation | Gaussian | log(verbascoside) concentration ~ Subregion + Population + (1\|Maternal Family) |
| calceolarioside B | linear mixed model | log transformation | Gaussian | log(calceolarioside B) concentration ~ Subregion + Population + (1\|Maternal Family) |
| mimuloside | linear mixed model | log transformation | Gaussian | log(mimuloside) concentration ~ Subregion + Population + (1\|Maternal Family) |
| unknown PPG 16 | linear mixed model | log transformation | Gaussian | log(unknown PPG 16) concentration ~ Subregion + Population + (1\|Maternal Family) |

Table S1: Model specifications for predicting phenylpropanoid glycoside (PPG) concentrations.

| **Region** | Coastal | Cordilleran | ENA | Northern | Southern | UK |
| --- | --- | --- | --- | --- | --- | --- |
| Coastal | NA | *0.066* | 0.181 | 0.243 | 0.400 | 0.517 |
| Cordilleran | NA | NA | 0.761 | 0.124 | *0.071* | **0.018** |
| ENA | NA | NA | NA | 0.555 | 0.211 | *0.080* |
| Northern | NA | NA | NA | NA | 0.393 | 0.112 |
| Southern | NA | NA | NA | NA | NA | 0.186 |
| UK | NA | NA | NA | NA | NA | NA |

Table S2: P-values from pairwise PERMANOVA tests to assess differences in phytochemical arsenal composition among individual biogeographic regions. Values in **bold** indicate pairwise comparisons that are significantly different (p < 0.05); values in *italics* indicate pairwise comparisons that are marginally significant.

| **Region** | Coastal | Cordilleran | ENA | Northern | Southern | UK |
| --- | --- | --- | --- | --- | --- | --- |
| Coastal | NA | 0.301 | 0.934 | 0.765 | 0.700 | 0.984 |
| Cordilleran | NA | NA | **0.026** | **0.002** | **0.016** | **0.001** |
| ENA | NA | NA | NA | **0.020** | 0.410 | 0.880 |
| Northern | NA | NA | NA | NA | *0.074* | **0.001** |
| Southern | NA | NA | NA | NA | NA | **0.015** |
| UK | NA | NA | NA | NA | NA | NA |

Table S3: P-values from pairwise PERMANOVA tests to assess differences in herbivore communities among individual biogeographic regions. Values in **bold** indicate pairwise comparisons that are significantly different (p < 0.05); values in *italics* indicate pairwise comparisons that are marginally significant.

| **Region** | Coastal | Cordilleran | ENA | Northern | Southern | UK |
| --- | --- | --- | --- | --- | --- | --- |
| Coastal | NA | 0.129 | **0.023** | *0.078* | 0.400 | **0.002** |
| Cordilleran | NA | NA | **0.021** | **0.014** | **0.046** | **0.001** |
| ENA | NA | NA | NA | **0.027** | *0.082* | **0.001** |
| Northern | NA | NA | NA | NA | 0.925 | **0.002** |
| Southern | NA | NA | NA | NA | NA | **0.001** |
| UK | NA | NA | NA | NA | NA | NA |

Table S4: P-values from pairwise PERMANOVA tests to assess differences in vegetation communities among individual biogeographic regions. Values in **bold** indicate pairwise comparisons that are significantly different (p < 0.05); values in *italics* indicate pairwise comparisons that are marginally significant.

| **Region** | Coastal | Cordilleran | ENA | Northern | Southern | UK |
| --- | --- | --- | --- | --- | --- | --- |
| Coastal | NA | **0.016** | **0.025** | **0.008** | 0.100 | **0.003** |
| Cordilleran | NA | NA | **0.011** | 0.441 | **0.023** | **0.001** |
| ENA | NA | NA | NA | *0.055* | **0.026** | **0.001** |
| Northern | NA | NA | NA | NA | 0.441 | **0.001** |
| Southern | NA | NA | NA | NA | NA | **0.001** |
| UK | NA | NA | NA | NA | NA | NA |

Table S5: P-values from pairwise PERMANOVA tests to assess differences in overall climate among individual biogeographic regions. Values in **bold** indicate pairwise comparisons that are significantly different (p < 0.05); values in *italics* indicate pairwise comparisons that are marginally significant.

**SUPPLEMENTARY FIGURES**


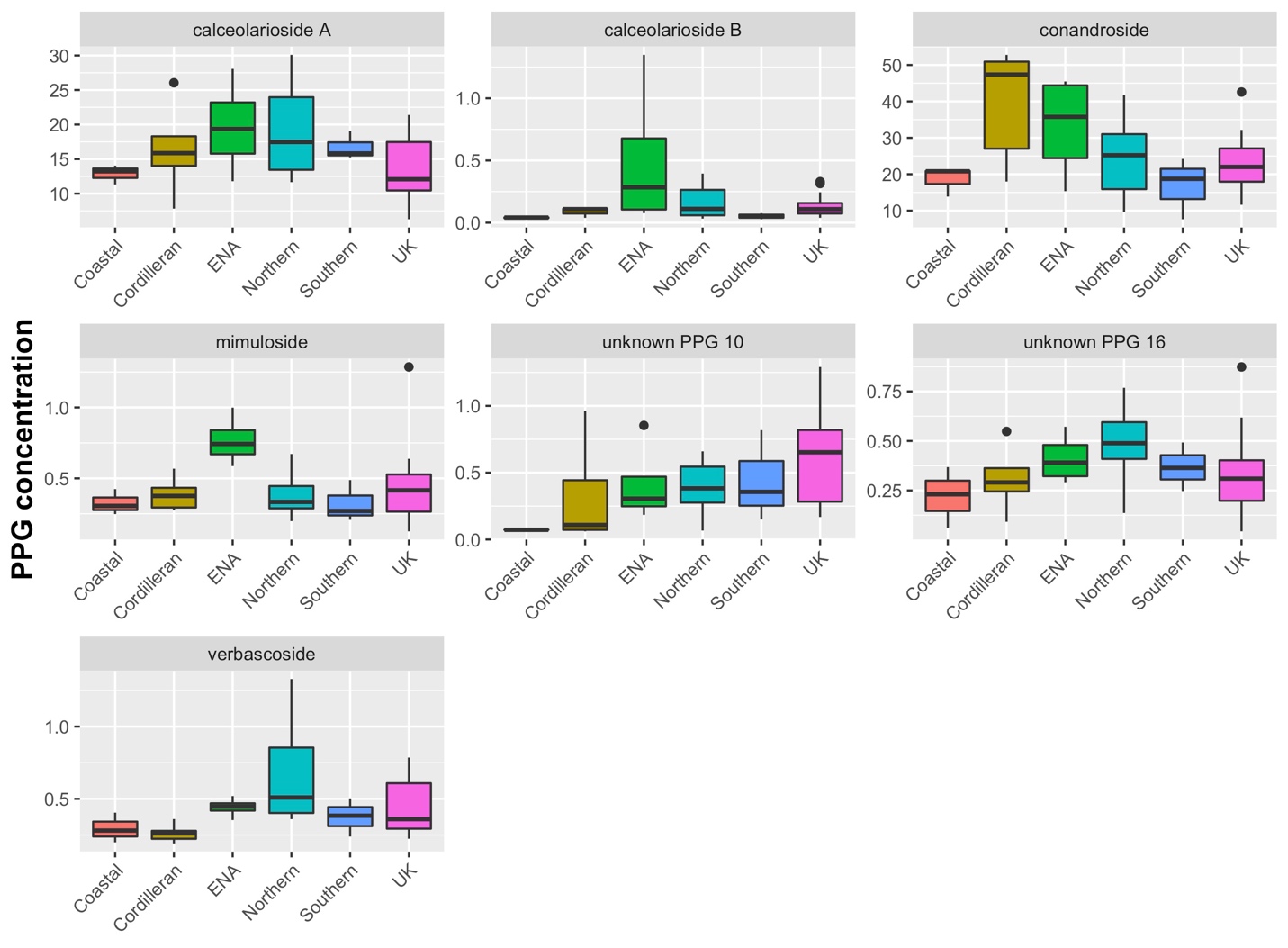


Figure S1. Boxplots of predicted total (summed) phenylpropanoid glycoside (PPG) concentrations (mg/g dry plant mass) for 41 yellow monkeyflower populations from 6 biogeographic regions. We quantified total PPG concentration in multiple individuals (n = 3 on average) from multiple maternal families (n = 4 on average) from each population. These results summarize population-level model predictions from our linear mixed models and generalized linear mixed models for PPG concentration.

**
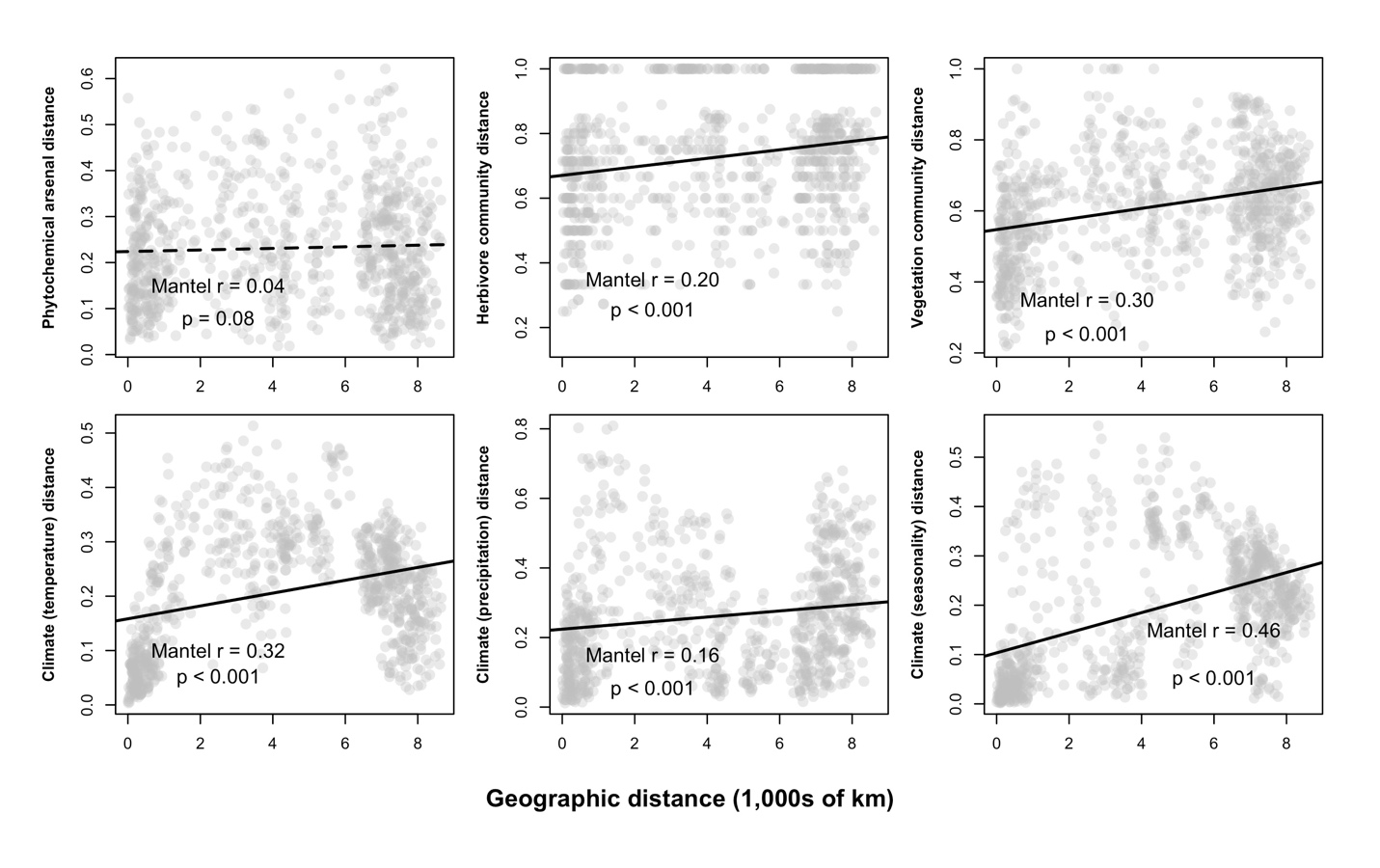
**

Figure S2: Relationship between physical distance and A) phytochemical arsenal dissimilarity; B) herbivore community distance; C) vegetation community distance; D) climate distance based on temperature variables; E) climate distance based on precipitation variables; and F) climate distance based on seasonality variables.


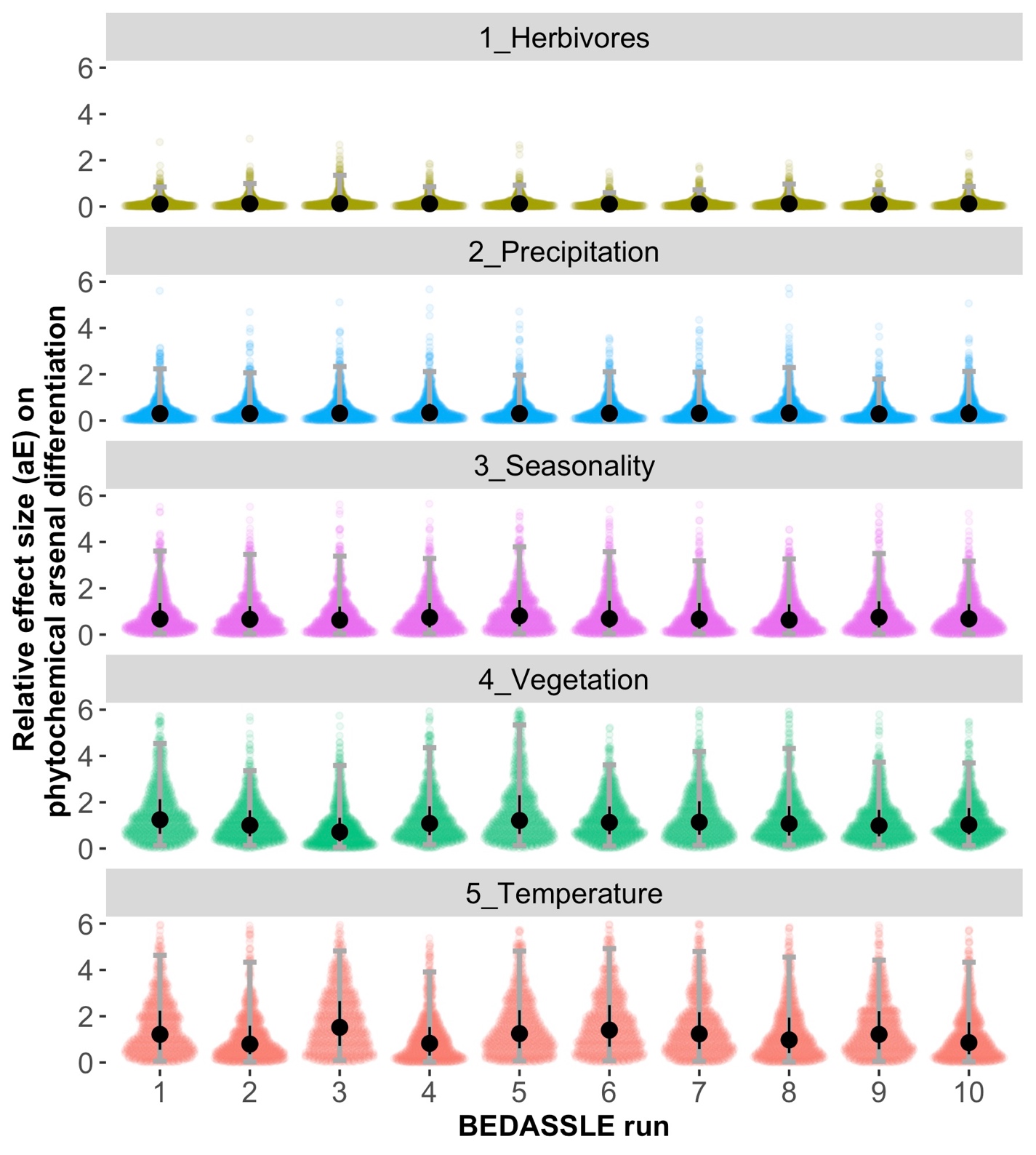


Figure S3: Comparison of effect sizes (aE) for each of five predictors of phytochemical resistance arsenal differentiation across 10 independent BEDASSLE MCMC runs. For all five potential predictors, 95% credible intervals for parameter estimates (gray error bars) as well as interquartile ranges (black error bars) overlapped in all runs, indicating that the models always converged on the same regions of highest probability. Points represent median effect size estimates from each independent run.


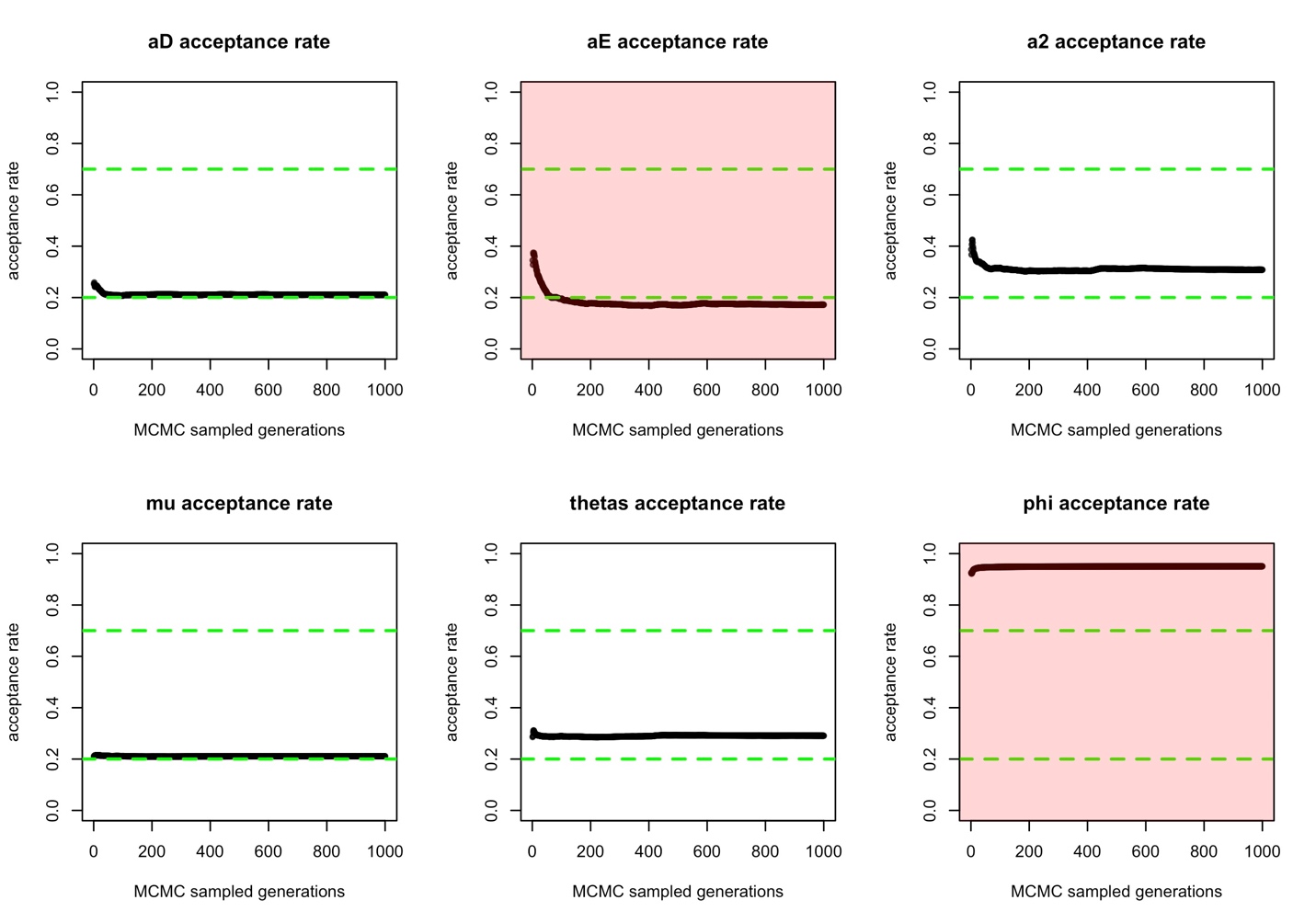


Figure S4: Acceptance rates from one randomly selected BEDASSLE MCMC sampling run. Acceptance rates between 20% and 70%, demarcated by horizontal green lines, produce optimal mixing behavior of the MCMC sampling chain. Post burn-in plateaus indicate convergence on stationary distributions. Tinted backgrounds indicate that acceptance rates are outside of optimal thresholds, which we further diagnosed by initiating multiple independent MCMC runs and determining whether parameter estimates from independent runs converged.


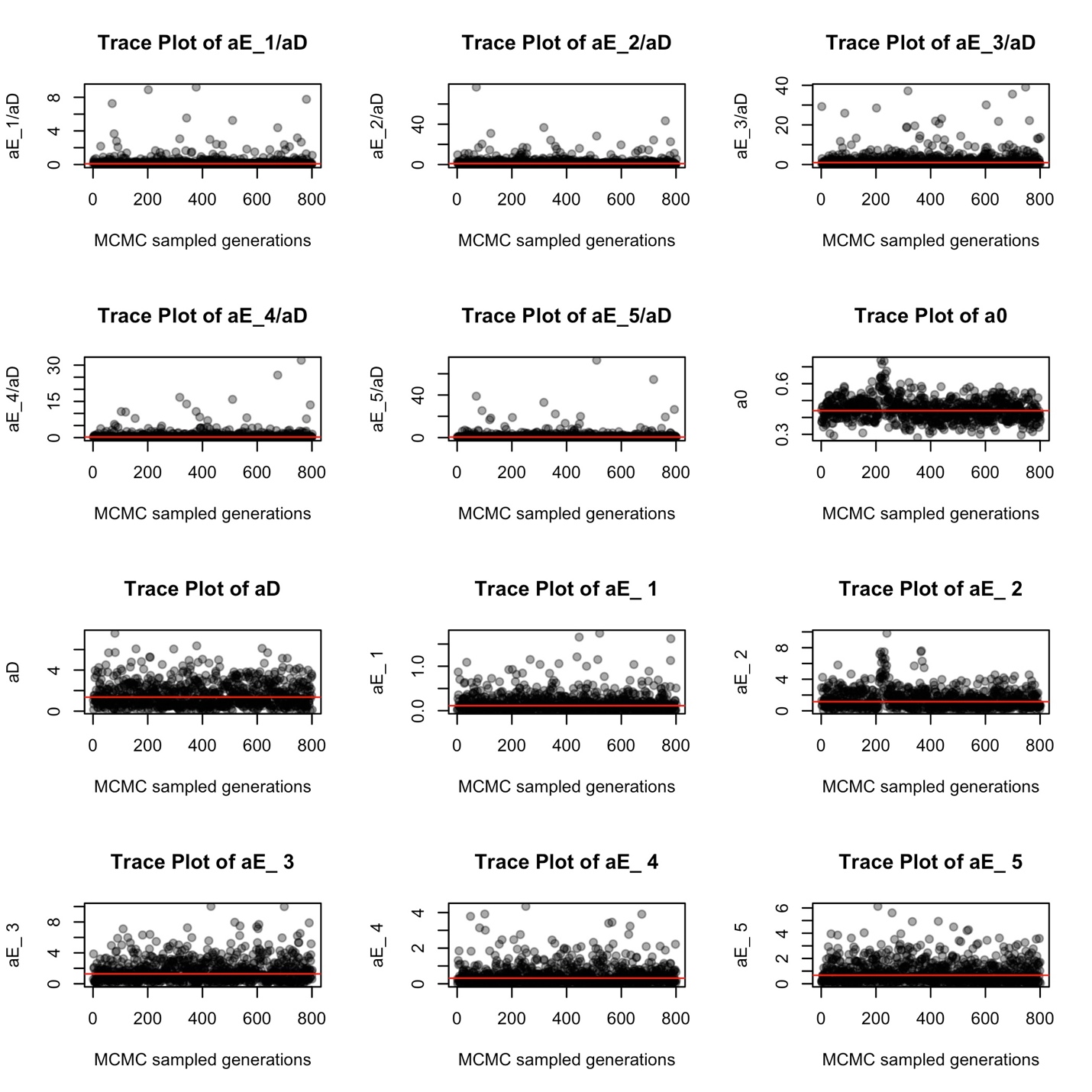


Figure S5: Trace plots from one randomly selected BEDASSLE MCMC sampling run. Appropriate chain mixing is indicated by parameter estimates that remain relatively constant across MCMC generations, resulting in “fuzzy caterpillar” shapes. Trace plots of chief parameters of interest indicated good model performance.


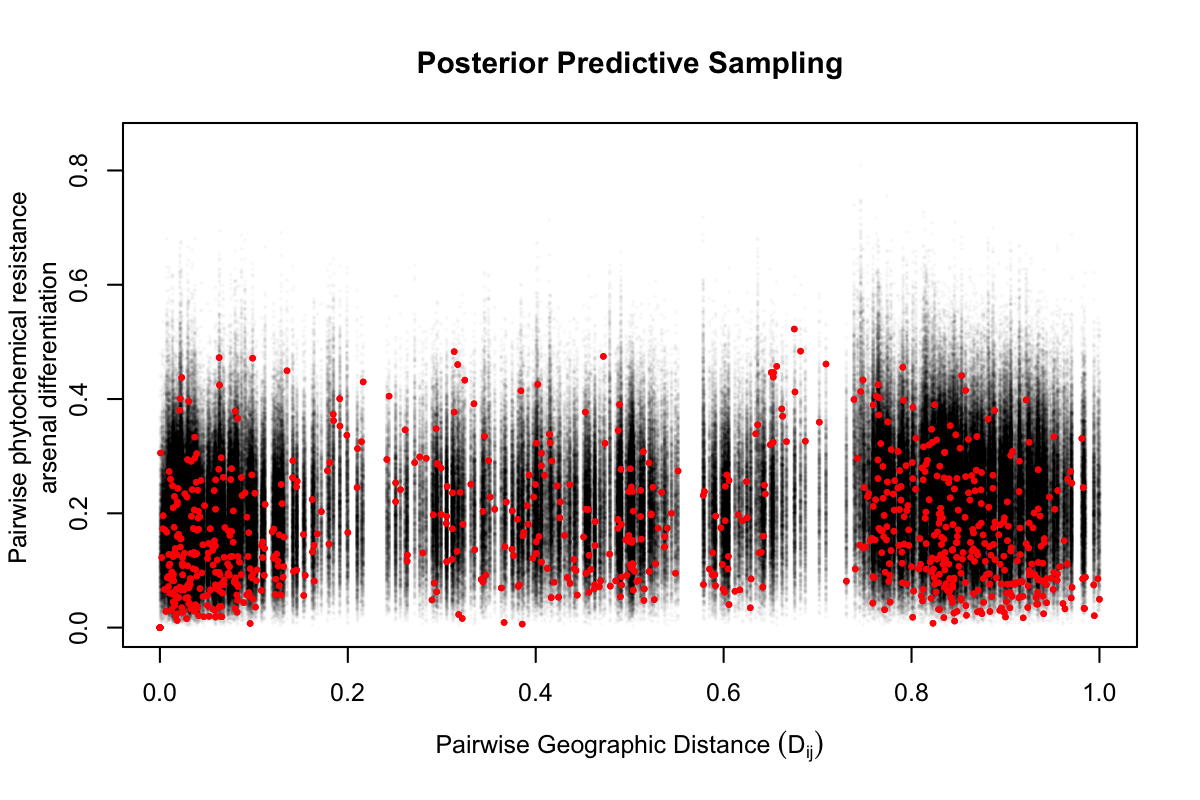


Figure S6: Posterior predictive sampling from one randomly selected BEDASSLE MCMC sampling run. Red points represent observed pairwise differentiation of resistance arsenals among 41 populations of *M. guttatus*, and black points represent simulated pairwise values. Overlap between simulated and observed arsenal differentiation indicates that the Bayesian model fits the data

**Appendix S1.**

BEDASSLE model parameters can be interpreted as the relative effect of a 1-unit change in a biotic or abiotic predictor on the differentiation of phytochemical arsenal composition. BEDASSLE is designed to use raw allelic count data (e.g., the “A” allele is present in 7 out of 10 individuals sampled), so we converted chemical concentrations (mg/gram of dry plant tissue as measured in the lab) to represent “counts” standardized based on the maximum concentration observed for each PPG. We then input a pairwise distance matrix (D) and five ecological distance matrices (E) consisting of pairwise herbivore, vegetation, and climatic distances into the *MCMC_BB* function in the *BEDASSLE* package in R (Bradburd 2013). We ran a beta-binomial model for overdispersion (i.e., some populations of *M. guttatus* differ more from the global mean phytochemical arsenal composition than others), estimating model parameters using MCMC simulations of 10 million generations, sampling from the posterior distribution every 10,000 generations. We removed the first 20% of the posterior as burn-in, allowing the simulation to reach a stationary distribution. We assessed MCMC performance by viewing post burn-in acceptance rates and trace plots and conducted posterior predictive sampling following the recommendations of BEDASSLE (Bradburd et al. 2013).

We initiated ten independent MCMC runs to determine whether 95% credible intervals for model parameters overlapped in independent runs. We amalgamated data from all MCMC runs and extracted post-burn effect sizes (i.e., the relative effect of each potential ecological driver on the differentiation of phytochemical composition) and summarized median, interquartile ranges, and 95% credible intervals for each predictor. BEDASSLE was designed to model pairwise F_ST_ based on unlinked loci as a joint function of physical and ecological distances (Bradburd et al. 2013). Phenylpropanoid glycosides (PPGs) might violate this assumption as they might be correlated due a shared biosynthetic pathway; however, such a pattern does not appear to be pervasive, as PPG production was only correlated in ~18% of pairwise comparisons involving 12 different PPGs in a comparative analysis across 21 *Mimulus* species (Holeski et al. 2021). Given this potential violation, we also further explored potential correlations in PPG production in *M. gutattus* using the *rcorr* function from the *Hmisc* package (Harrell 2021). Among the seven PPGs we assayed, we found that 11 of 21 pairwise comparisons showed significant correlations (average Pearson’s r = 0.4), indicating moderate correlation among about half of the PPGs.

Ninety-five percent credible intervals derived from post-burn-in MCMC posteriors overlapped for all five of the predictor variables; however, this seems to be driven by abundant data points at the near-zero end of the distribution (Fig. 5), as there were substantial differences in median effect sizes and inter-quartile ranges among predictors (Fig. 5). Overall, 95% credible intervals for individual effect sizes (aE) from all 10 independent MCMC runs overlapped, indicating that the models had converged on the region of highest probability for each of the parameter estimates (Fig. S2). Further diagnosis of the BEDASSLE MCMC sampling algorithm indicated good performance as acceptance rates fell mostly within acceptable ranges (Fig. S3). Trace plots of parameters of interest suggested convergence on stationary distributions (Fig. S4), and posterior predictive sampling plots indicated a general agreement between observed and simulated data (Fig. S5).

**Appendix S2.**

Instead of modeling genetic similarity at unlinked sites, we modeled phytochemical arsenal similarity based on the concentration of seven phenylpropanoid glycosides (PPGs) that may share a common biosynthetic pathway (Holeski et al. 2021). We observed a moderate correlation (Pearson’s r = 0.4) between the concentrations of about half of the PPG pairs, clearly violating the assumption that our loci (i.e., PPGs) were unliked. This violation of the BEDASSLE model assumptions itself is not expected to bias median parameter estimates, but it may result in narrow 95% credible intervals (Grieneisen et al. 2019). Next, BEDASSLE was intended to model responses resulting from spatially homogeneous processes, such as those under mutation-migration balance (Bradburd et al. 2013). Here for example, multiple waves of colonization to the UK (Vallejo-Marín et al. 2021) may have resulted in adjacent populations that have greater phytochemical differentiation than expected based on their physical distance. That said, for population genetic models in general, and for BEDASSLE specifically, few natural populations will conform to all model assumptions, and the model can still perform robustly under heterogeneous generating processes (Bradburd et al. 2013), similar to those that may have shaped phytochemical resistance arsenal differences in populations of *M. guttatus*.

BEDASSLE returns effect sizes for ecological predictors in the same units as the input matrices, so care is required in comparing effect sizes for different predictors. We calculated both climate, herbivore, and vegetation distance matrices all based on Bray-Curtis similarity, therefore individual model parameters are easily comparable. A Bray-Curtis distance of one indicates a complete lack of overlap, or complete turnover of community members or climatic conditions across multiple bioclimatic variables. In data from our field surveys, we had both herbivore and vegetation communities that shared no community members, and therefore had Bray-Curtis distances equal to one (Fig 4). Interpreting how a 1-unit change in these predictors affects resistance arsenal differentiation makes biological sense, since our dataset includes such communities. Conversely, the greatest pairwise climatic distance among any of the population pairs in our dataset was between 0.5-0.8 depending upon the component of climate (Fig. 4). A 1-unit change in climatic space is biologically unrealistic for the *M. guttatus* populations that we studied, even for the populations in our dataset experiencing the most disparate climates. Thus, the very large effect size of temperature on defensive arsenal similarity among *M. guttatus* populations (Fig. 5) may only be about half as strong in nature for the populations in our dataset.
